# Sorting efficiency in mechanical sorting of construction and demolition waste

**DOI:** 10.1177/0734242X20914750

**Published:** 2020-04-28

**Authors:** Marko Hyvärinen, Mikko Ronkanen, Timo Kärki

**Affiliations:** 1 Fiber Composite Laboratory, Lappeenranta University of Technology, Finland; 2 Destaclean Oy, Finland

**Keywords:** Construction and demolition waste, mechanical sorting, separation, material recycling, waste

## Abstract

The requirements for the recycling of construction and demolition waste are tightening, and companies in the waste business have improved their performance to comply with new legislation. Construction and demolition waste includes various recyclable materials, such as metals, plastics and wood. However, effective material recovery requires functional and reliable technology for sorting in order to separate waste fractions into useful secondary materials or applications. This paper concerns the mechanical sorting efficiency of construction and demolition waste with a commercial mechanical sorting equipment consisting of a roller screening and an air separation unit. Sorting ability is studied with pre-sorted and crushed test material. Sieve analysis of pre-sorted test material is used to define particle size distribution before sorting. The quality criteria of construction and demolition waste vary greatly, depending on a number of factors which pose a challenge to the operation of the sorting system. The studied sorting equipment was found to be reliable for producing nine different fractions from pre-sorted and crushed material mixtures. The requirements for the purity level of the fractions and the profitability of utilization define the number of fractions to be sorted cost-efficiently. Typically, a compromise between cost and purity level has to be found.

## Introduction

Increasing concern about the environment, as well as tightened environmental laws, have forced companies in the waste business to improve and encourage the recycling of wastes. Material recycling and the utilization of wastes reduce the production costs, environmental pollution and the use of non-renewable (oil-based) resources, as well as improve the properties of materials (Bledzki et al., 2010; Hyvärinen and Kärki, 2015; Koleva et al., 2011; La Mantia and Morreale, 2008; Nourbakhsh and Ashori, 2010; Panthapulakkal and Sain, 2007; Srivastava and Maurya, 2015).

Construction and demolition waste (CDW) is formed at different stages of the construction/demolition process of individual residences, commercial buildings and other civil engineering structures (Huang et al., 2002). CDW represents one of the most voluminous waste streams generated in the European Union (EU), accounting for approximately 30% of all waste generated in the EU (Eurostat, 2016), and it differs both quantitatively and qualitatively by EU countries. The EU has set binding legislation, according to which 70% by weight of non-hazardous CDW must be prepared for reuse, recycled or recovered by 2020. Also the amount of biodegradable or organic materials in landfilling will be reduced to a maximum of 10% by weight (European Commission, 2010). To meet these tightened regulations, new methods for CDW recycling have to be developed (Väntsi and Kärki, 2014). This includes also the development of material sorting and processing during recycling processes.

CDW consist of various recyclable materials, such as metals, plastics, wood, gypsum, mineral wool, cardboard and paper, and concrete. CDW is generally considered to be harmless to the environment, and thus landfill has been commonly used. Nowadays, due to identification of environmental hazards and detection of the value of recycling, research is focused on the recycling and reuse of materials. Studies have shown that approximately 90% of CDW can be recycled, which reduces the need for landfill sites. CDW also includes fractions such as asbestos and chromated copper arsenate (CCA)-impregnated wood that cannot be transported to landfill sites because they require treatment according to the law. Furthermore, contamination of building materials, for example with tar, peat, oil or paint, can result in special requirements for recycling (Christensen, 2011). Typically, the waste fractions generated on new construction sites are often less different and less contaminated, and therefore have higher recycling potential than the waste fractions of demolition sites, which are often more contaminated, mixed and even contain hazardous materials. These differences set challenges for the mechanical sorting of CDW.

Currently, a variety of sorting processes in CDW recycling are applied in different geographic areas in Europe. However, the technological basis of the sorting systems remains the same in the design of the different processes. CDW sorting technologies are based on two levels of technology according to the degree of waste separation. On one hand, simple technology based on automated crushing machinery that consists typically of feed hoppers, crushing systems and magnetic separators, together with various screens and conveyors can be used. On the other hand, more complex technology can be utilized to process highly mixed waste streams (Vegas et al., 2015).

For further utilization of recycled material, the waste material has to be sorted to meet the required levels of purity. Sorting criteria may be the size, magnetism, density, conductivity and colour of particles, as well as particle size or moisture content. The sorting of mixed CDW requires different kinds of technologies linked together to generate an efficient sorting process (Tchobanoglous and Kreith, 2002; Vegas et al., 2015). Typically, factors such as the operation cost, desired purity level, capacity and space requirements for the machinery specify the applicable technology for CDW sorting (Mulder et al., 2007).

## Materials and methods

In this research, CDW material received from two separate locations, named as Stream1 (S1) and Stream2 (S2), of a local recycling company is studied. S1 represents waste load from the renovation site of an old apartment building, and S2 represents a construction site of a new building. The waste materials were pre-sorted manually, and classified into nine fractions: (a) paper and board, (b) gypsum, (c) concrete and porcelain, (d) mineral wool, (e) wood, (f) metal, (g) plastic, (h) undefined, and (i) fines.

After pre-sorting, the mass of crushable fractions, that is, plastic, wool, board, wood and gypsum, was measured, and metals and other unwanted materials, such as polystyrene and polyvinylchloride plastics, were removed. The proportion of each fraction separated in the material mixture was determined and expressed as a percentage by weight.

The selected test fractions were crushed with an Untha LR630 single-shaft shredding system equipped with a ram system that pushes the material against the rotor with replaceable cutting inserts. The sieve opening of the shredder was 20 mm, and the rotor speed 98 rpm. The crushed fractions were weighed individually, and then mixed. The bulk density of the material mixture was determined after mixing.

Sieve analysis was carried out with a Retsch AS200 Basic vibratory sieve shaker. Of both material mixtures, specimens that exemplified the composition of the mixture were selected for sieving. The sieving time was three minutes. The mesh diameters were 5.60, 2.80, 2.00, 1.40, 1.00, 0.71, 0.50, 0.315 and 0.212 mm. The particle size distribution of the material mixture was obtained as the result of the sieving analysis.

**Figure 1.**
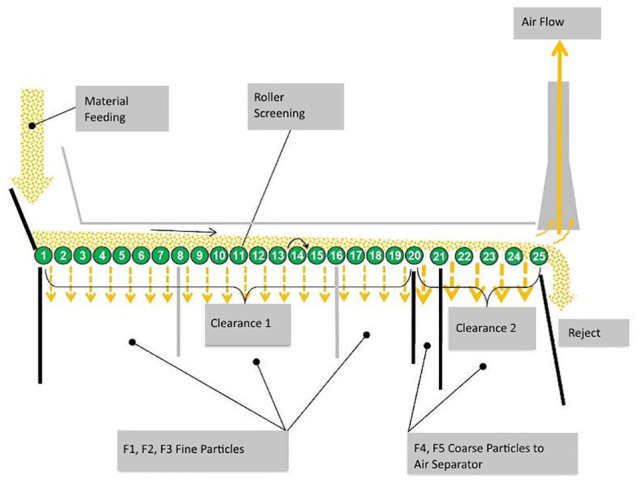
Functional principle of the commercial sorting equipment.

Mechanical separation of the pre-sorted material mixture was performed with commercial sorting equipment which consisted of a roller screening and an air separator unit. The material mixture was sorted to nine fractions; seven of them were separated with a laboratory-sized screening unit and two with an air separator. The parameters used in the separation were based on previously presented values generally used in the sorting of CDW. The roller screening unit consisted of 25 rolls. Figure 1 shows the principle of the sorting technology. The separation ability of the air separator was controlled by adjusting the air flow rate with a potentiometer. The separated particles were named as ‘ac’ and ‘rej’. The term ‘ac’ described particles flowing upwards, while the term ‘rej’ described particles falling down against the air flow.

## Results and discussion

Before sorting, CDW materials S1 and S2 were manually pre-sorted and classified into nine different material fractions. The constituents of the pre-sorted fractions by weight are shown in Table 1. Of the pre-sorted fractions, plastic, wool, board, wood and gypsum were selected for mechanical sorting. Before sorting, these fractions were weighed and crushed individually. Table 2 summarizes the measured weights and proportions of the total weight of the fractions before crushing.

**Table 1.** Constituents of CDW material after pre-sorting.

Fraction	S1 (wt%)	S2 (wt%)
Metal	22.7	20.7
Plastic	15.8	15.2
Wood	14.4	13.7
Wool	8.1	11.4
Fine	6.8	6.2
Concrete	3.6	1.2
Gypsum	3.2	0.5
Board	1.9	0
Undefined	23.5	31.3

**Table 2.** Distribution of selected waste fractions before crushing.

	Wood	Plastic	Wool	Gypsum	Board	Total
S1 (kg)	26.2	19.2	17.3	6.9	3.6	73.1
	36%	26%	24%	9%	5%	100%
S2 (kg)	34.2	22.6	10.3	0.8	25.1	93.1
	37%	24%	11%	1%	27%	100%

The total weight of material mixture S1 was 73.1 kg with the bulk density of 142.37 kg m^−3^ and moisture content of 5.7%. Material mixture S2 had the weight of 93.1 kg, bulk density of 94.03 kg m^−3^ and moisture content of 5.4%. The distribution of wood was the highest in both mixtures, 36% and 37%. Also, plastic had a fairly equal proportion in both mixtures, at 26% and 24%. Board had the greatest variation in the material mixtures. The distribution of board was 5% in S1 and 27% in S2. The difference can be explained by the fact that in a new construction site, a lot of the building materials are covered with board packaging.

Sieve analysis was carried out to define the particle size distribution of the material mixtures. The amount of sieved material of mixture S1 was 54.5 g, and of mixture S2 31.5 g. Table 3 presents the particle size distribution determined by sieving. The research findings indicate that the particle size distribution of both streams present quite a similar gross pattern, excluding oversized particles.

**Table 3.** Particle size distribution of the material mixtures.

Mesh size	#5.60	#2.80	#2.20	#1.40	#1.00	#0.71	#0.50	#0.315	#0.212	Over
S1 (wt%)	53.33	21.27	7.30	4.44	3.17	0.63	1.27	1.27	0.95	5.40
S2 (wt%)	41.28	19.63	7.52	5.69	4.04	1.47	2.02	1.28	1.47	14.13

The material mixtures S1 and S2 were mechanically sorted with roller screening and an air separator. The sorting test was repeated four times for S1 (named as A–D) and seven times for S2 (named as A–G). The results of the mechanical separation process by percentages are shown in Tables 4 and 5. The separated fractions F1–F3 were classified as fines. Fractions F4 and F5 were fed to the air classifier, where these fractions were sorted into lighter particles F4ac and F5ac, and heavier particles F4rej and F5rej. The measured density of each separated fraction is presented in Table 6.

**Table 4.** Fraction distribution of test material mixture S1, in percentage (wt%).

	F1	F2	F3	F4	F5	F6	F7	Fines	F4ac	F4rej	F5ac	F5rej
A	12.19	4.33	1.54	48.20	22.25	3.46	8.03	18.06	17.05	29.14	12.36	8.31
B	10.06	4.31	1.56	48.39	24.97	3.34	7.36	15.94	18.17	29.67	15.05	9.55
C	9.85	4.31	1.56	46.34	23.11	5.98	8.85	15.72	17.82	26.96	14.03	8.59
D	10.64	4.19	1.50	50.57	24.49	2.12	6.49	16.33	19.06	31.05	15.22	9.08
SUM	10.69	4.29	1.54	48.37	23.71	3.73	7.68	16.49	18.01	29.15	14.17	8.89
St. Dev.	0.92	0.06	0.03	1.50	1.09	1.40	0.87	0.92	0.72	1.47	1.14	0.48

**Table 5.** Fraction distribution of test material mixture S2, in percentage (wt%).

	F1	F2	F3	F4	F5	F6	F7	Fines	F4ac	F4rej	F5ac	F5rej
A	8.48	2.37	0.77	37.26	28.80	2.88	19.44	11.62	29.34	5.45	19.26	4.73
B	7.73	2.52	0.94	36.57	29.60	4.79	17.85	11.19	31.78	4.50	25.10	4.54
C	8.39	2.48	1.11	44.04	24.98	3.28	15.73	11.98	38.12	5.35	23.87	3.83
D	7.14	2.25	0.86	35.87	30.43	5.12	18.32	10.25	31.21	4.60	25.78	4.55
E	7.30	2.27	0.82	36.54	30.45	4.00	18.62	10.39	31.15	4.93	25.40	4.78
F	7.04	2.48	0.96	34.63	29.89	5.39	19.60	10.48	30.06	4.08	25.65	3.68
G	7.05	2.21	0.79	35.14	29.72	4.64	20.44	10.05	30.34	4.09	26.01	3.86
SUM	7.59	2.37	0.89	37.15	29.13	4.30	18.57	10.82	31.71	4.71	24.44	4.28
St. Dev.	0.58	0.12	0.11	2.93	1.77	0.88	1.41	0.69	2.72	0.51	2.21	0.44

**Table 6.** Density of the separated fractions.

	F1	F2	F3	F4rej	F4ac	F5rej	F5ac	F6	F7
S1 (kg m^−3^)	154.0	94.5	95.5	331.3	106.3	170.1	60.9	65.0	41.2
S2 (kg m^−3^)	190.9	117.9	93.5	291.4	124.0	232.3	81.8	53.6	29.8

The repeatability of mechanical sorting with the sorting equipment and air separator was at a good level, and significant deviations between the results of various test runs were not observed. This indicates that the material mixture had uniform quality, and the machine parameters were adjusted correctly for the applied material flow. Improved sorting efficiency and high purity levels were the aims of the parameter adjustment. In adjusting the air flow rate of the air separator, the goal was to sort the particles into different fractions by weight.

As a result of the mechanical sorting with the sorting equipment, nine fractions were separated from both test material mixtures. Fractions F1–F3 represented fine particles consisting mainly of small wool fibres, but also including small wood fibres and gypsum dust. The bulk density of the fine particles was observed to be at a relatively high level. As a dense and heavy material, the use of small wool fibres could be studied as reinforcing or filler material, for example in concrete pouring. Cheng et al. (2011) studied the use of mineral wool in concrete-based composites, and the results showed that the addition of mineral wool improved the compression strength, splitting tensile strength, absorption power and resistivity significantly, and improved wear resistance slightly.

Fractions F4 and F5 were directed to the air separator where fluffy and light particles, such as wood, plastic and board particles, which can be utilized in composites or energy production, were redirected to fractions F4ac and F5ac. Heavier, denser and larger particles, for example hard plastic, wood and gypsum particles, headed to fractions F4rej and F5rej, which are the most valuable ones for further utilization because of their hard plastic particles content. Therefore, these fractions should be purified more thoroughly to improve the result of the separation process and the utilization of plastics.

For particle size-based separation, it might be contemplated to use for example a vibrating screen or a drum screen, where stick-shaped wood particles penetrate through the sieve mesh while plastic particles continue moving on towards the reject fraction. Also, the shape of gypsum particles differs considerably from plastic particles. Gypsum particles are generally small and circular in shape. They also sink in water, while plastics and wood remain on the surface of water. The separation of plastic and wood particles could be studied also by using eddy current separation. In addition to sieve or eddy current separation, one alternative method could be sensor-based separation, if this kind of sorting can be considered economically viable.

The type of wool affects its particle size when crushed. Mineral wool decomposes to smaller particle size than glass wool. Material mixture S1 consisted mostly of glass wool, whereas material mixture S2 contained mainly mineral wool. In mechanical sorting, this influenced separation accuracy, because mineral wool with a smaller particle size ended up mainly in fines, while glass wool particles were found from several fractions (i.e. fines, F5ac, F6 and F7). On the other hand, due to the higher quantity of wool in mixture S1 than S2, the effect of wool type was slightly difficult to define.

Overall, the separation of different fractions from a CDW mixture with sorting equipment is reasonable if the sorted fractions can be reclaimed profitably. The purity level of the separated fractions can be affected easily with the control system of the equipment. An important observation is the fact that the hard plastics could be separated effectively from the rejects. However, in the case of hard plastics, the purity level of the reject fraction was only about 50%, so further purification of the hard plastics fraction would be needed, depending on the requirements of the intended further application. Another important observation is that the gypsum particles could be almost perfectly separated to fines. Only the larger sized particles of gypsum ended up as rejects.

The roller screening and air separator unit as a mechanical separation system for mixed CDW has not been studied earlier. Instead, the use of air jigging in the separation of mixed CDW is researched in several studies. Studies showed that the batch jigging separation was technically capable of removing undesired contaminants from mixtures of concrete and brick. Contamination levels of less than 1% in mass and approximately 90% in mass of pure concrete were reached for all studied cases (Ambrós et al., 2017). Air jigging separation for removing concrete and brick particles from recycled CDW aggregates was found to result in a purity level of 95 wt% (Hu et al., 2019). In removal of low-density materials, such as wood and paper, air jigging has potential to reach separation performances comparable to those of air classifiers and automatic sorting systems. The results also pointed out that the initial content of contaminants does not have a significant effect on the separation extent (Ambrós et al., 2017). Also, the use of heavy liquid separation in the separation of gypsum and organic material from CDW has been studied. Heavy liquid separation as a wet process with the use of hazardous chemicals can be utilized with the density range in which gypsum/organic material was concentrated. Also, it was pointed out that in practical separation application, a dry separation process that can produce separation at this high-density range would be preferable (Montero et al., 2010).

## Summary

Due to tightening environmental laws, material recycling and waste reduction will become more and more important in the future. The utilization of recycled materials decreases the need for virgin materials, as well as reduces the environmental impact of wastes. Effective and reliable mechanical sorting technologies for CDW are an essential part of material recycling and recovery. In this study, the applicability of one mechanical sorting equipment arrangement was examined.

CDW consists typically of various recyclable materials, such as wood, metals and plastics. However, continuous changes in the composition of CDW due to the different building materials used in different time periods and collected in different regions create challenges for sorting.

The studied mechanical sorting equipment was found to be suitable for the mechanical sorting of CDW. The sorting equipment was able to separate different fractions from pre-sorted material mixtures. Techno-economically, the number of fractions to be separated depends on the profitability of utilization and the purity level requirements for these fractions. For economic reasons, it is not generally appropriate to produce the highest purity level of fractions. In certain cases, some impurities are acceptable, for example a mixture of wood and thermoplastic is suitable for manufacturing composite materials.

As concerns the present study, it has to be taken into account that the studied CDW material mixture did not include hazardous or unrecognized materials. Therefore, further research with actual waste material is required. Also, in order to improve the sorting efficiency, further purification of the separated fractions might be needed. Operation capability in real life is currently unexplored, because commercial sorting equipment is not yet used in the field of CDW sorting.
